# Late-Onset Seizure Disorder in Adult Cerebral Palsy Associated With COVID-19 Infection

**DOI:** 10.7759/cureus.37438

**Published:** 2023-04-11

**Authors:** Caroline S McCauley, Vivian Li, Steven Kobrin

**Affiliations:** 1 Medical School, Lake Erie College of Osteopathic Medicine, Erie, USA; 2 Internal Medicine, St. John's Episcopal Hospital, Far Rockaway, USA

**Keywords:** seizure disorder, covid-19 neurologic deficits, cerebral palsy (cp), cytotoxic lesion, covid-19

## Abstract

COVID-19 can affect many organ systems, including the CNS, with symptoms of altered mental status and seizures. We present a case of a 30-year-old man with cerebral palsy who developed seizures after a COVID-19 infection. Admission labs were remarkable for hypernatremia, and elevated creatine kinase, and troponin levels as well as creatinine above baseline. MRI was performed demonstrating a small, evolving acute/subacute abnormality in the midline splenium of the corpus callosum. An EEG showed moderate to severe abnormalities with low-voltage delta waves. The patient was treated with medication and advised to follow up with a neurologist. One month later, no residual CT abnormality corresponding to the previously reported lesion in the midline splenium of the corpus callosum was observed. Although epilepsy is a common finding in patients with cerebral palsy, the complete lack of seizure activity throughout this patient's early life, coupled with previously unremarkable brain imaging, further supports our claim that his recent onset of seizures was directly related to COVID-19. This case highlights the possibility of new seizures in patients with pre-existing neurological conditions after COVID-19 infection and emphasizes the need for more research.

## Introduction

The novel COVID-19 (SARS-CoV-2) has caused widespread disruption for the past three years, leaving many questions unanswered. Some patients continue to suffer from the effects of 'Long COVID' where symptoms persist after the resolution of the acute infection. In some cases, these symptoms may include rare neurological complications. Kincaid et al. report a patient with 'Long COVID' returning to the hospital six days following his discharge with new onset seizure activity reportedly unexplained by any other causes [1]. Hence, it is crucial to recognize the possibility of various neurological manifestations related to COVID-19 infections.

Herein we present a case of a 30-year-old black male with cerebral palsy who developed seizures shortly after a COVID-19 infection. Epilepsy is common among patients with cerebral palsy; however, it typically manifests within the first two years of life. Thus, here we suggest that 'Long COVID' may likely be the cause of new-onset seizures in this patient. The purpose of our report is to increase awareness of a rare but potentially debilitating complication and provide information on the diagnosis and management of COVID-19-related seizures.

## Case presentation

A 30-year-old black male with cerebral palsy and previous hospitalization for COVID-19 presented to the ED on post-discharge day five due to focal tonic-clonic seizure with impaired awareness. On the previous visit, a CT head was performed for evaluation of acute encephalopathy, which was negative for any acute intracranial abnormality. The patient was later discharged with a single dose of Paxlovid.

He then returned to the ED five days later with reports of continued high-grade fevers (101-103 °F) refractory to antipyretic agents and subsequent tonic-clonic seizures associated with tongue biting. Seizure episodes reportedly lasted 20 minutes and were bilateral tonic-clonic movements occurring in the upper extremities. Seizure activity was controlled with 5 mg midazolam administered by EMS en route to the hospital.

On arrival at the ED, the patient was postictal, febrile to 104 °F, and tachycardic. Admission labs were remarkable for hypernatremia, creatine kinase, and troponin levels. Serum creatinine was also noted to be above baseline (Table 1). Urine toxicology done in the ED was negative. Repeat COVID-19 polymerase chain reaction (PCR) serology was negative and lumbar puncture was not done due to no meningeal signs being appreciated on the physical exam. The patient was admitted to the telemetry unit for evaluation of new-onset seizures and rhabdomyolysis.

**Table 1:** Comprehensive metabolic panel from both hospital admissions. Abnormal values are marked as *. BUN: Blood urea nitrogen, LDH: Lactate dehydrogenase, eGFR: Estimated glomerular filtration rate, AST: Aspartate transaminase, ALT: Alanine transaminase, PT: Prothrombin time, INR: International normalized ratio, PTT: Partial thromboplastin time, CK: Creatine kinase

	Normal Values	COVID-19 Admission	Seizure Admission
Sodium	135-145 mmol/L	142 mmol/L	156* mmol/L
Potassium	3.5-5.1 mmol/L	4.5 mmol/L	3.7 mmol/L
Chloride	98-107 mmol/L	101 mmol/L	117* mmol/L
Bicarbonate	22-32 mmol/L	25 mmol/L	30 mmol/L
BUN	8-25 mg/dL	15* mg/dL	27* mg/dL
Creatinine	0.6-1.1 mg/dL	0.8 mg/dL	1.3* mg/dL
Glucose	70-105 mg/dL	118 mg/dL	115 mg/dL
Lactic acid	0.4-2.0 mmol/L	3.7* mmol/L	1.9 mmol/L
Calcium	8.4-10.5 mg/dL	10 mg/dL	9.3 mg/dL
Phosphorus	2.8-4.5 mg/dL	3.4 mg/dL	4.7* mg/dL
Magnesium	1.6-2.6 mg/dL	2.0 mg/dL	2.7* mg/dL
Ferritin	24-336 mg/L	77 mg/L	
LDH	140-280 units/L	543* units/L	
eGFR	60-137 mL/min	137.34 mL/min	78.43 mL/min
Total Bilirubin	0.2-1.4 mg/dL	0.6 mg/dL	1.3 mg/dL
AST	7-21 U/L	36* U/L	148* U/L
ALT	7-55 U/L	29 U/L	72* U/L
PT	10-14 seconds	13.6 seconds	
INR	1.0	1.19	
PTT	32–45 seconds	25.9 seconds	
D-Dimer	0-229 ng/mL	209 ng/mL	
CK	21–215 U/L		6216* U/L
Troponin I	0.00-0.034 ng/mL		0.036* ng/mL

**Figure 1:**
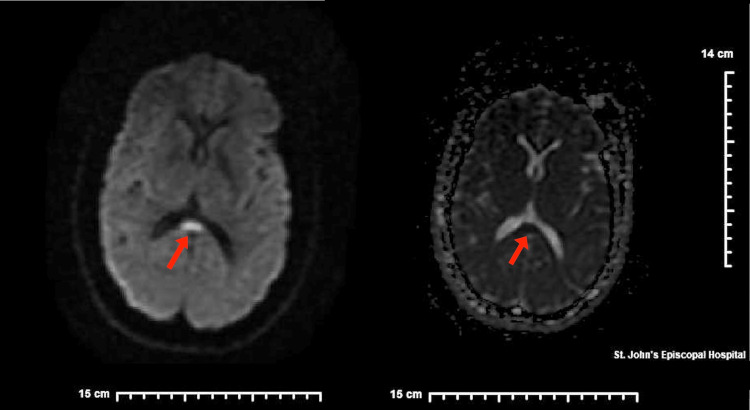
MRI T2-weighted axial DWI images from seizure admission containing lesions in the corpus callosum marked with red arrows. DWI: Diffusion-weighted imaging

MRI was performed demonstrating a small evolving acute/subacute abnormality in the midline splenium of the corpus callosum without other evidence of a recent infarct (Figure 1). EEG was notable for moderate to severe abnormalities with low voltage delta waves (Table 2). Neurology was consulted and the patient was started on oxcarbazepine 600 mg twice daily. The rest of the hospital course was uneventful, and the patient was later discharged with a neurology clinic follow up.

**Table 2:** EEG report table demonstrating moderate/severe abnormal findings and disorganized low voltage activity. Cps: Cycles per second

Waves	Frequency (cps)	% of Time	EEG Pattern
Alpha Waves	Rare, Normal (8-13)	Rare	None observed
Beta Waves	15-20,Normal (14-30)	30-40%	Diffuse, symmetrical
Theta Waves	4-6, Normal (4-8)	40-50%	Diffuse, symmetrical
Delta Waves	1-3, Normal (0.5-4)	30-40%	Low voltage

**Figure 2:**
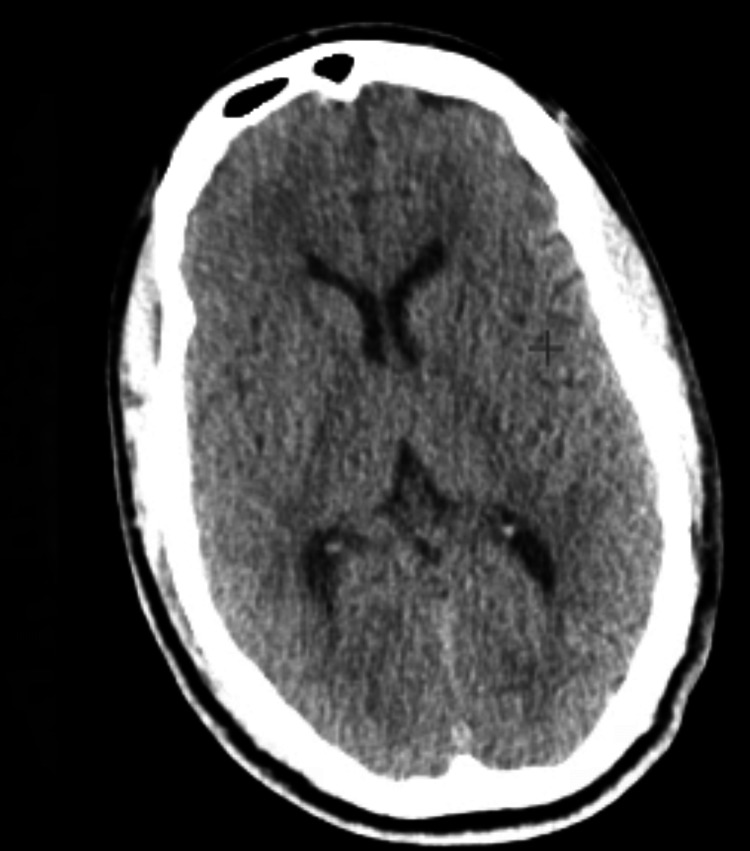
Brain CT from one month following seizure showing normal neuroimaging.

He returned to the ED one month later for evaluation of headaches. There was no residual CT abnormality appreciated corresponding to the previously reported abnormality of the midline splenium of the corpus callosum suggesting a transient cytotoxic lesion (Figure 2). 

## Discussion

This case details the potential for new-onset seizures in a patient with a pre-existing neurological condition following a COVID-19 infection. In this patient with cerebral palsy but no previous seizure history, it is possible that the increased metabolic demands from COVID-19 may have lowered the seizure threshold. Our patient presented to the ED with hypernatremia (156 mmol/L) which can lead to lethargy, weakness as well as seizures in severe cases as the serum sodium concentration approaches > 158 mmol/L. In our patient in the context of a prior COVID-19 infection with continued high-grade fevers (101-103 F), hypernatremia may be occurring secondary to dehydration. However, in a case series of 12 critically ill COVID-19+ patients, hypernatremia (151-159 mmol/L) was found to persist throughout the ICU stay but was not correlated with severe neurological symptoms [2]. Kasim et al, proposed a possible mechanism for this hypernatremia suggesting that the SARS-COV-2 virus can bind to the ACE2 receptors found on the kidney, resulting in increased resorption of sodium resorption in the proximal convoluted tubule [3]. Our report adds to previous findings, such as those by Moriguchi et al. and Vollono et al, which describe a similar seizure presentation following a COVID-19 infection, leading to metabolic abnormalities in previously healthy patients or patients with well-controlled epilepsy [4,5].

Previously reported cytotoxic lesions of the corpus callosum (CLOCC) have occurred in various medical conditions, including infections, trauma, and malignancy [6]. In two pediatric COVID-19+ patients, CLOCC was directly linked to a cytokine storm and multisystem inflammation. Increasing inflammatory cytokines in the blood and cerebrospinal fluid can lead to vasodilation, creating edema in myelin and disrupting the blood-brain barrier-ultimately causing seizures [7,8]. Chougar et al. previously reported that while reviewing the neuroimaging findings of COVID-19 patients with neurologic symptoms, 53% of patients had MRI abnormalities ranging from cerebrovascular lesions to CLOCCs [9].

Additionally, the transient nature of cytotoxic lesions of the corpus callosum (CLOCC) increases the risk for a misdiagnosis of cerebrovascular accident (CVA)/transient ischemic attack (TIA) and subsequent treatment with medications which may increase bleeding risk [10]. To make an accurate diagnosis, serial cerebral imaging is indispensable, and findings must correspond with the patient’s clinical presentation. In the present case, our patient only had one brain MRI done during admission and a subsequent brain CT scan was done a month later. The brain CT scan done on follow-up for our patient is not as sensitive to the evaluation of CLOCCs and a brain MRI is the preferred image modality. Frequent neurological exams should be performed to monitor for any focal deficits. In some cases, empiric anticonvulsant therapy may be warranted for patients with a high risk (>60%) of a second seizure due to underlying neurological conditions [11].

Individuals with cerebral palsy have a significantly higher risk of developing epilepsy when compared to the general population, with seizure rates reaching 20%-45% in children with CP [12]. In these cases, the first seizure is 50%-60% more likely to occur within the first year of life [12]. Neuroimaging of children with CP demonstrates significantly less brain volume, as well as changes in the basal ganglia and periventricular leukomalacia [12]. The complete lack of seizure activity throughout this patient's early life, coupled with previously unremarkable brain imaging, further supports our claim that his recent onset of seizures was directly related to a COVID-19 infection. 

## Conclusions

The above case report further emphasizes the need for continued research involving atypical presentations of COVID-19. COVID-19 has the ability to affect many organ systems including the CNS and can manifest with symptoms of altered mental status and/or seizures. Initial diagnostic testing may include cerebral imaging modalities such as MRI or CT of the head, as well as EEG, and cerebrospinal fluid analysis. Diagnostic testing can help to establish patient prognosis and guide further therapy. Although glucocorticoids and immunomodulatory agents have been proven to reduce mortality in patients with dyspnea and supplemental oxygen use, their benefits in the management of COVID-19 encephalopathy remain unclear.

While prior studies have explored the potential association between cytotoxic lesions in the corpus callosum and COVID-19, our patient's condition was further complicated by cerebral palsy. The symptoms of COVID-19 in neurologically impaired patients may vary, and it is important for physicians to be mindful of these changes as they can influence future treatment plans. More information is needed to better understand and determine the neurologic impact of COVID-19 infection. 
